# Effects of Exercise on Frailty in Older People Based on ACSM Recommendations: A Systematic Review and Meta-Analysis of Randomized Controlled Trials

**DOI:** 10.3390/jcm13113037

**Published:** 2024-05-22

**Authors:** Neng Pan, Zbigniew Ossowski, Jun Tong, Dan Li, Shan Gao

**Affiliations:** 1 Faculty of Physical Culture, Akademia Wychowania Fizycznego I Sportu, 80-336 Gdansk, Poland; zbigniew.ossowski@awf.gda.pl; 2 Department of Sport, Kunming Medical University, Kunming 650000, China; tongjun080511@163.com; 3 Academy of Sport, Yunnan Normal University, Kunming 650000, China; 17279123996@163.com (D.L.); gaoshan199608@163.com (S.G.)

**Keywords:** frailty, elderly, ACSM recommendations, exercise intervention, exercise dose

## Abstract

**Objectives:** The objective of the study was to carry out an analysis of the methodological quality of clinical trials (effects of exercise on frailty in older people) based on ACSM recommendations. **Methods**: The search scope included PubMed, Embase, Web of Science, Cochrane, and literature that cannot be retrieved from the database. The topic was the impact of exercise on frailty in elderly people. Changes in five outcome measures (FP, BI, SPPB, GS, and BMI) were assessed using mean differences (MD) and 95% confidence intervals (95% CI). A random effects model (RE) was used to conduct a meta-analysis and compare the results between subgroups. **Results:** The intervention effects of exercise on the five outcome indicators of frailty in elderly people were all significant (*p* < 0.05). The effect of a high-consistency subgroup on outcome indicators FP and GS was more significant than that of the low- or uncertain-consistency subgroup (MD: −1.09 < −0.11, MD: 2.39 >1.1). There was no significant difference in the intervention effect as reflected in the outcome measures SPPB and BMI in the high-consistency subgroup (*p* = 0.07, *p* = 0.34). There was no significant difference in the impact of the intervention on the outcome measure BI between the two subgroups (*p* = 0.06, *p* = 0.14). **Conclusions:** Exercise prescriptions with high consistency with ACSM recommendations may be more effective in both FP and GS interventions than those with uncertain or low consistency. However, it is essential to note that the data derived from the meta-analysis is still subject to the small number of studies, the unknown degree of consistency of participants in individual studies, and the different mix of cases in the studies.

## 1. Introduction

The clinical syndrome that meets three or more of the following criteria is referred to as frailty: unexpected weight loss (10 pounds in the last year), poor physical activity, self-reported tiredness, grip strength weakening, and sluggish walking [1]. The clinical condition known as frailty is defined by an individual’s extreme susceptibility to both internal and external stimuli [2]. The world’s aging population is one of the demographic groups seeing the most drastic changes. Current estimates indicate that between 2015 and 2030, there will be 1.4 billion persons worldwide who are 60 years of age or older, up from 901 million in 2015. By 2050, that number is expected to approach 2.1 billion [3]. Frailty in elderly people is a multi-dimensional syndrome that involves the interaction of biological, psychological, and social factors. It is associated with a higher risk of adverse outcomes, such as a decline in functional ability, falls, delirium, institutionalization, hospitalization, and death [4,5]. Exercises, dietary intervention, multi-component treatments, and individually customized geriatric care models are the four main kinds of interventions that have been tried to enhance the health outcomes of weak patients or, more recently, to battle frailty [6]. Many macronutrients and micronutrients have been shown to either directly cause or interact with frailty, indicating that diet plays a crucial role in both avoiding and exacerbating frailty syndrome [7]. However, more longitudinal studies on this topic are required to further understand the potential role of nutrition in preventing, postponing, or reversing frailty syndrome [8]. In elderly patients, frailty and polypharmacy are prevalent and well-researched conditions, but little is known about how they could affect one another [9]. Reducing polypharmacy could be a cautious strategy to prevent and manage frailty. Further research is needed to confirm the possible benefits of reducing polypharmacy in frailty development, reversion, or delay [10]. Low-level care could be promoted as a primary intervention [11]. Exercise reduces age-related oxidative damage and chronic inflammation, increases autophagy, and improves mitochondrial function, the myokine profile, the insulin-like growth factor-1 (IGF-1) signaling pathway, and insulin sensitivity [12]. Consequently, physical activity and exercise are regarded as one of the primary methods for preventing the physical deterioration associated with frailty in elderly people.

Lifestyle behaviors like physical activity can help manage frailty levels [13]. Frailty is not a reason to avoid physical activity; in fact, it can be one of the most significant reasons to recommend it [14]. Although the optimal level of exercise intervention intensity (duration and frequency) is yet unknown, consistency is consistently high across different programs [4]. Physical performance tests have been used as alternative measures of frailty since they are associated with, or predictive of, frailty [15,16,17]. Although the Short Physical Performance Battery (SPPB) was originally designed to assess lower limb function, it has also been used to gauge physical frailty in earlier research [18,19]. Grip strength is a viable test to administer in a clinical context and has been utilized as a single item measure for frailty in several investigations [16,20]. One of the most used frailty assessments, the Fried Frailty Phenotype (FP), operationalizes frailty as a biological phenotype into five quantifiable criteria [1]. The Barthel Index is a valid measure of disability [21]. These are important predictors of health outcomes and are therefore useful outcome measures to assess the effectiveness of exercise. A person’s height and weight are used to calculate their body mass index (BMI), which enables them to be categorized as overweight or obese [22]. Body fat contributes to the association between BMI and frailty, and a larger proportion of body fat is linked to frailty [23]. All of the indicators mentioned above can be affected by exercise. For older persons who are at risk of frailty, exercise is the medication that may reverse or alleviate frailty, maintain quality of life, and restore independent functioning [24].The best approach for enhancing gait, balance, and strength in older adults while also lowering their fall risk and preserving their functional ability as they age appears to be a multi-component exercise intervention program that includes strength, endurance, and balance training [25]. Nonetheless, some academics have suggested that in order to choose the most beneficial exercise regimen, additional research on this subject that also includes fragile populations is required [26].

The American College of Sports Medicine (ACSM) has created exercise regimens that are advised for older folks. These regimens include specific recommendations for the dosage of cardiorespiratory exercise, resistance training, and balancing exercise for frail people [27,28]. However, it is currently unclear whether exercise interventions based on the ACSM recommendations will significantly impact frailty in elderly people more than exercise interventions with low or uncertain consistency. This systematic review aims to analyze the methodological quality of clinical randomized controlled trials (effects of exercise on frailty in older adults) based on ACSM recommendations.

## 2. Materials and Methods

The Preferred Reporting Items for Systematic Reviews and Meta-Analyses (PRISMA) statement have been followed in reporting the systematic review and meta-analyses, and will be registered in PROSPERO (CRD42024517899).

### 2.1. Search Strategy

Our search strategy was based on the PICOS principle. We searched the PubMed, Embase, Web of Science, and Cochrane databases from their inception to 20 January 2024. It was last searched on 25 January 2024. It focused on disease type, study population, intervention, and research methodology. The search terms included the following: (“Asthenia” or “Frailty” or “Fatigue” or “Neurasthenia” or “Muscle Weakness” or “Frailties” or “Frailness” or “Frailty Syndrome” or “Debility” or “Debilities”) AND (“Exercise” or “walking” or “Nordic Walking” or “Exercises” or “Physical Activity” or “Activities, Physical” or “Activity, Physical” or “Physical Activities” or “Exercise, Physical” or “Exercises, Physical” or “Physical Exercise” or “Physical Exercises” or “Exercise, Aerobic” or “Aerobic Exercise” or “Aerobic Exercises” or “Exercises, Aerobic” or “Exercise Training” or “Exercise Trainings” or “Training, Exercise” or “Trainings, Exercise” or “Training, Resistance” or “Strength Training” or “Training, Strength” or “Balance” or “Ambulation” or “Stair Climbing” or “Walking, Nordic” or “Pole Walking” or “Walking, Pole”) AND (“Randomized controlled trial” or “controlled clinical trial” or “randomized” or “placebo” or “randomly”) AND (“aged” or “elderly”). The detailed search strategy is shown in Supplementary Materials. We manually searched for the literature that could not be retrieved from the database. When necessary, we contacted the authors by email for further information.

### 2.2. Criteria for Selection of Studies

First of all, we need to state that there are no restrictions on the publication time and language of the included articles. The study inclusion criteria were as follows: (a) published studies using randomized controls (RCTs); (b) the participants consisted of older adults who had previously received a diagnosis of pre-frailty or frailty, as well as those exhibiting suspected symptoms of frailty (either during hospitalization or shortly after discharge). Different clinical trials have used different criteria to identify frailty in patients. Some trials have used a Barthel Index score of 50 or higher and the MEC-35, which is a modified and validated version of the mini-mental state test in Spanish. Other trials have used a walking pace test and a chair standing test to identify frailness. Some trials have even used patients’ self-reports and the number of falls they have experienced as criteria for determining frailty. These methods are able to derive participants in a state that approximates, but is not exactly equivalent to, pre-frailty or frailty; (c) any kind of exercise program, such as resistance, flexibility, or aerobic training, might be used as an intervention; (d) the control group only received routine care that did not include exercise or carried out daily life; (e) outcome measures included at least one of five outcomes related to frailty in elderly people: grip strength, BI, SPPB, the Fried scale, and BMI.

The research exclusion criteria included the following: (a) reports, meeting minutes, comments, etc., were not considered; (b) in the intervention group, interventions that combined exercise, nutrition, drug treatment, etc.; (c) in the control group, in addition to routine care and daily life, all interventions; (d) redundant experimental data that appeared in many papers related to the same research were excluded.

The titles and abstracts of the literature that satisfied the inclusion requirements were separately examined by two writers (N.P. and J.T.). The whole text of the paper was retrieved if one of the writers determined that a research paper satisfied the criteria. Subsequently, two writers separately evaluated whether the whole text satisfied the requirements. In the event that an agreement could not be reached, debate led to the decision being taken by the fourth author (D.L.). Subjects were defined as older individuals without regard to language, body mass index, gender, or publication date constraints.

### 2.3. Data Synthesis and Analyses

Two authors (N.P. and J.T.) independently extracted data for the included studies. Grip strength and Fried scale were the primary outcomes; SPPB, BI, and BMI were the secondary outcomes. An Excel spreadsheet was designed in advance to extract the relevant data, including publication characteristics (author name, country, publication year), methodological characteristics (interventions, sample size), participant characteristics (age, gender), campaign characteristics (intervention period, intervention frequency, duration), risk assessment, and outcome characteristics.

When extracting the result data, Engauge Digitizer 4.1 software was used to extract the research data presented in the form of pictures. We extracted data only immediately after the intervention for studies with multiple follow-up assessments.

After the data extraction, the exercise intervention was evaluated for dose and consistency. The exercise intervention doses in this study were assessed against ACSM recommendations for developing and maintaining cardiorespiratory, muscle, skeletal, and neurological function in healthy adults [29]. According to ACSM recommendations, two authors (NP and JT) independently evaluated each study’s exercise intervention based on different criteria defined for exercise dose (frequency, intensity, duration, etc.) to assess consistency to exercise dose (Table 1).

The scoring criteria for each indicator in this meta-analysis was 0 points for not completely meeting the standards; 1 point for not being sure whether it meets or may meet the standards; 2 points for completely meeting the standards. If two authors had different opinions during the review process, a third author was invited to discuss this until a consensus was reached. This scoring rubric was used to calculate the proportion of each metric that meets the ACSM recommended exercise measures. When the proportion was ≥70%, this meant a high-consistency relationship with ACSM recommendations; when <70%, this meant a low- or uncertain-consistency relationship with ACSM recommendations.

### 2.4. Biased Risk Assessment

Two pairs of authors completed quality assessments (NP and JT and ZO, DL, and SG). The evaluation tool used was the Cochrane risk of bias tool (Rob). The evaluation reference standard was Cochrane Collaboration’s tool for assessing the risk of bias [30]. This study’s investigations were all randomized controlled trials. Evaluation indicators include incomplete outcome data (attrition bias), biased reporting (reporting bias), blinding of personnel and participants (performance bias), allocation concealment (selection bias), random sequence generation (selection bias), and other bias [30]. Three categories were used to categorize bias risk: “low risk,” “unclear risk”, and “high risk”.

### 2.5. Statistical Analyses

This meta-analysis used REVIEW MANAGER 5.4.1 to perform statistics and analysis on the data, divided into two groups: high consistency with ACSM guidelines and low or uncertain consistency with ACSM guidelines. In the heterogeneity test within the subgroup, if I^2^ > 50%, the random effects model was used, and if I^2^ < 50%, the random effects model or the fixed effect model was used [31]. When the included literature used scales to evaluate outcome indicators, if the scales used were different, standard mean difference for analysis was used; if the scales used were the same, mean difference for analysis was used.

## 3. Results

### 3.1. Study Selection

**Figure 1:**
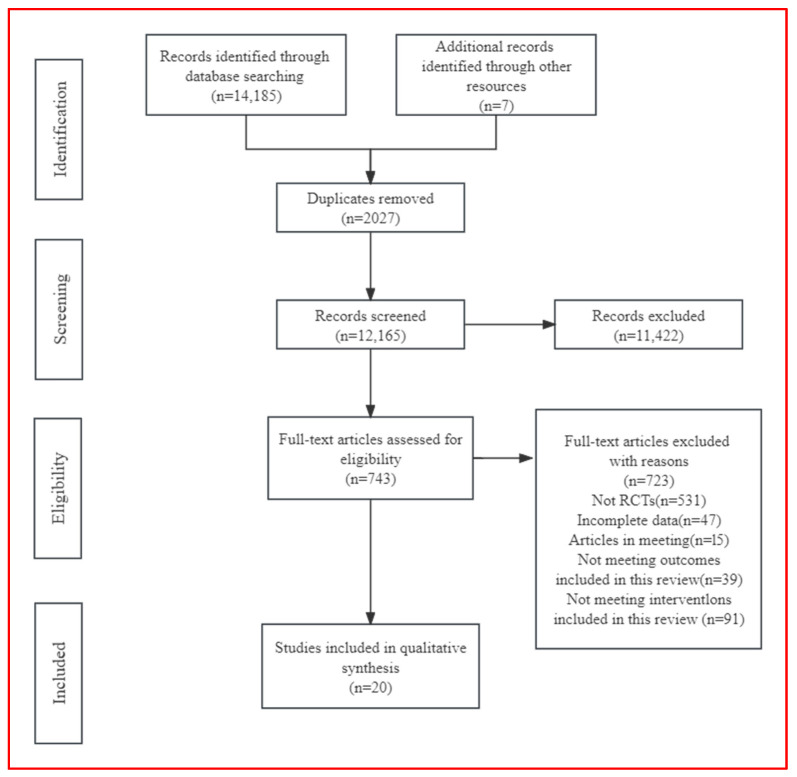
PRISMA study flow diagram.

The retrieved literature included PubMed (2027), Embase (45), Web of Science (3712), and Cochrane (8401), totaling 14,185 articles. A total of seven articles were found through other search methods. After removing duplicates (2027), 12,165 articles remained. After a preliminary review of titles and abstracts, 743 articles remained; after a final evaluation of whole texts, 20 remained [32,33,34,35,36,37,38,39,40,41,42,43,44,45,46,47,48,49,50,51] (Figure 1).

### 3.2. Study Characteristics

In total, 2016 persons over 60 were enrolled in the 20 investigations (1004 in the intervention group and 1012 in the control group). The studies that were considered include the following outcome indicators: “BMI,” “grip strength,” “SPPB,” “Fried,” and “BI.” There are thirteen articles on “grip strength” result indicators, six on “Fried” outcome indicators, eight on “BI” outcome indicators, eleven on “SPPB” outcome indicators, and three on “BMI” outcome indicators [32,33,34,35,36,37,38,39,40,41,42,43,44,45,46,47,48,49,50,51]. The included articles are from France, Turkey, Spain, Denmark, Japan, Taiwan, Singapore, Ireland, Canada, Thailand, and China (Table 2).

The intervention periods in the included literature ranged from 8 to 32 weeks, ranging from 1 to 7 days per week, and the duration of a single intervention ranged from 5 to 90 min. Interventions included regular exercise training, individualized physical activity programs, multi-component physical exercise, dance, and Tai Chi. The intervention measures in the control group were daily life without participating in any physical exercise (Table 2).

Among the included literature, 14 studies were about cardiopulmonary exercise, 18 were about resistance exercise, and 11 were about flexibility exercise. Among them, a total of 10 articles have high consistency to ACSM recommendations (consistency ≥70%), and the remaining ten articles have low or uncertain consistency (consistency <70%) (Table 3).

### 3.3. Risk of Bias

**Figure 2:**
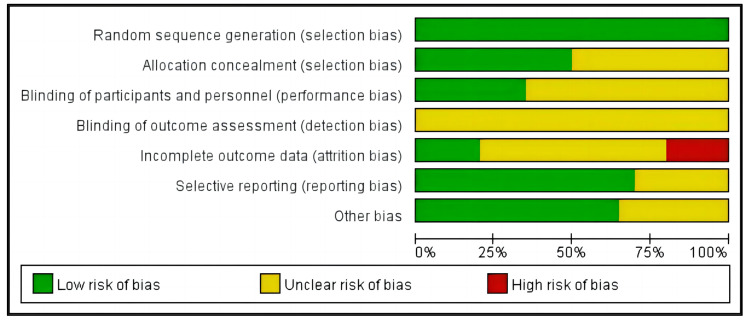
The risk of bias graph displays the percentages representing the review authors’ assessments of each risk of bias item for all the included research.

Low risk of bias >50%, include three indicators: bias in reporting (selective reporting), bias in random sequence generation (selection bias), and additional biases. Three signs indicate an unclear risk of bias ≥50%: allocation concealment (selection bias), staff and participant blinding (performance bias), and outcome assessment blinding (detection bias). Incomplete outcome data (attrition bias) present an unclear risk of bias and a high risk of bias ≥75%, as well as a high risk of bias ≈25% (Figure 2).

**Figure 3:**
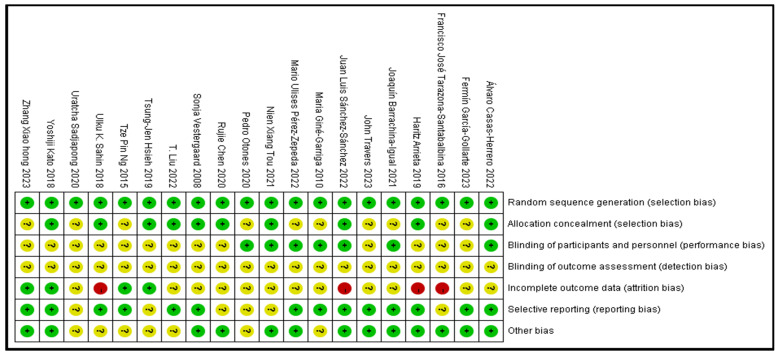
Review the authors’ assessments of each risk of bias item for every research article that is included in the risk of bias summary [32,33,34,35,36,37,38,39,40,41,42,43,44,45,46,47,48,49,50,51]. Red: there is a risk. Yellow: it is uncertain whether the risk exists. Green: there is no risk.

All 20 included articles were randomized controlled trials. Ten articles did not report their allocation methods. Only 13 articles used one blinding method because exercise intervention is difficult to implement under double-masked conditions. None of the 20 articles reported whether blinding was used when processing the results. Experimental samples were lost in 16 articles (12 articles lost samples <10 people, and 4 articles lost samples >10 people). Six articles have the possibility of selective reporting. It was unclear whether there were other risks of bias in seven articles (Figure 3).

### 3.4. The Impact of Consistency with ACSM Recommendations on the Fried Frailty Phenotype

Outcome indicator 1 (Fried Frailty Phenotype) contains a total of six articles, with 300 people in the intervention group and 306 people in the control group; there was high consistency with ACSM guidelines in five articles, and low or uncertain consistency with ACSM guidelines in one article. After the heterogeneity test (I^2^ = 97%), the random effects model was used for statistical analysis. All articles used the same scale for this outcome indicator, so mean difference (MD) was used for statistics and analysis.

Data analysis showed that the overall impact of exercise on the Fried Frailty Phenotype (FP) was −0.93 (95% CI: −1.75, −0.1), which was significantly different (*p* = 0.03). This shows that exercise has a significant intervention effect on FP.

The results of the subgroup analysis showed that in the high-consistency group, the MD was −1.09 (95% CI: −2.01, −0.16) and I^2^ = 98%, which was significantly different (*p* = 0.02). This indicates that exercise prescription with high consistency to ACSM guidelines significantly affects the FP intervention.

The results of the subgroup analysis showed that in the low- or uncertain-consistency group, the MD was −0.11 (95% CI: −0.53, 0.31), which was not significantly different (*p* = 0.61). It is unclear whether exercise prescription with low or uncertain consistency to ACSM guidelines significantly influences FP.

**Figure 4:**
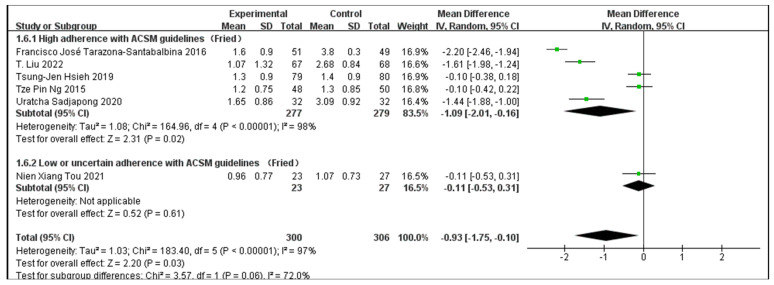
Forest plot for meta-analysis of the impact of exercise on FP indicators in frail elderly people [36,40,47,48,49,50]

In summary, exercise has a significant intervention effect on FP indicators; the MD was −0.93 (95% CI: −1.75, −0.1) and (*p* = 0.03). Exercise prescription with high consistency to ACSM guidelines has a more significant impact on FP than exercise prescription with low or uncertain consistency to ACSM guidelines (−1.09 < −0.11), and the intervention effect is better. Because the FP score is more significant, it indicates a higher degree of frailty [1] (Figure 4). However, the higher heterogeneity in the high-consistency subgroup may be due to the intervention period, intervention measures, and study sample characteristics (Table 2).

**Figure 5:**
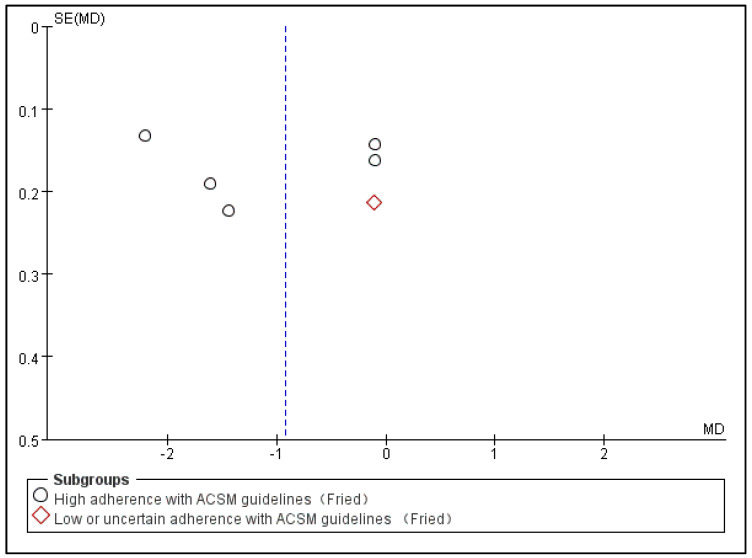
Funnel plot for FP indicator.

Subsequently, we conducted publication bias testing through REVIEW MANAGER 5.4.1. We observed the funnel plot and found that both sides were approximately symmetrical, indicating no obvious publication bias (Figure 5).

### 3.5. The Impact of Consistency with ACSM Recommendations on the Barthel Index

Outcome indicator 2 (Barthel Index) contains a total of eight articles, with 438 people in the intervention group and 432 people in the control group; there was high consistency with ACSM guidelines (four articles), and low or uncertain consistency with ACSM guidelines (four articles). After the heterogeneity test (I^2^ = 94%), the random effects model was used for statistical analysis. All articles used the same scale for this outcome indicator, so mean difference (MD) was used for statistics and analysis.

Data analysis showed that the overall impact of exercise on the Barthel Index (BI) was 5.79 (95% CI: 1.11, 10.46), which was significantly different (*p* = 0.02). This shows that exercise has a significant intervention effect on BI.

The results of the subgroup analysis showed that in the high-consistency group, the MD was 5.72 (95% CI: −0.16, 11.6) and I^2^ = 87%, which was not significantly different (*p* = 0.06). This indicates that it is unclear whether exercise prescription with high consistency to ACSM guidelines has a significant intervention effect on BI.

The results of the subgroup analysis showed that in the low- or uncertain-consistency group, the MD was 5.85 (95% CI: −1.95, 13.65), which was not significantly different (*p* = 0.14). It is unclear whether exercise prescription with low or uncertain consistency to ACSM guidelines has a significant intervention effect on BI.

**Figure 6:**
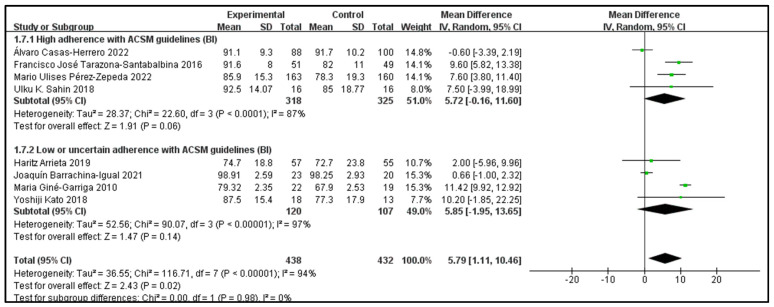
Forest plot for meta-analysis of the impact of exercise on BI indicators in frail elderly people [33,34,37,38,42,43,46,48].

In summary, exercise has a significant intervention effect on BI indicators; the MD was 5.79 (95% CI: 1.11, 10.46) and (*p* = 0.02). However, it is unclear whether exercise prescriptions with high consistency to ACSM guidelines or with low or uncertain consistency to ACSM guidelines have a significant intervention effect on BI indicators (Figure 6). The higher heterogeneity in the two subgroups may be due to the intervention period, intervention measures, and study sample characteristics (Table 2). It should be noted here that the higher the BI score, the stronger the independence and the lower the dependence.

**Figure 7:**
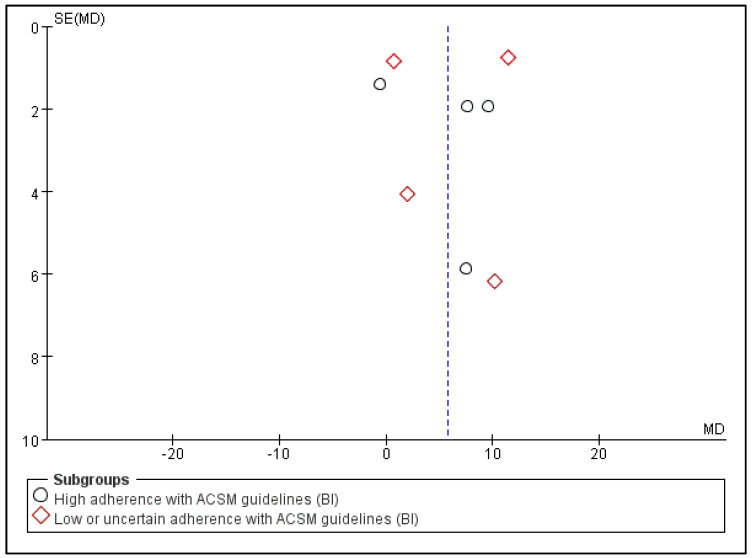
Funnel plot for BI indicator.

Subsequently, we conducted publication bias testing through REVIEW MANAGER 5.4.1. We observed the funnel plot and found that both sides were approximately symmetrical, indicating no obvious publication bias (Figure 7).

### 3.6. The Impact of Consistency with ACSM Recommendations on the Short Physical Performance Battery

Outcome indicator 3 (Short Physical Performance Battery) contains a total of 10 articles, with 585 people in the intervention group and 596 people in the control group; there was high consistency with ACSM guidelines in 4 articles, and low or uncertain consistency with ACSM guidelines in 6 articles. After the heterogeneity test (I^2^ = 93%), the random effects model was used for statistical analysis. All articles used the same scale for this outcome indicator, so mean difference (MD) was used for statistics and analysis.

Data analysis showed that the overall impact of exercise on Short Physical Performance Battery (SPPB) was 1.03 (95% CI: 0.28, 1.78), which was significantly different (*p* = 0.007). This shows that exercise has a significant intervention effect on SPPB.

The results of the subgroup analysis showed that in the high-consistency group, the MD was 1.42 (95% CI: −0.12, 2.96) and I^2^ = 95%, which was not significantly different (*p* = 0.07). This indicates that it is unclear whether exercise prescription with high consistency to ACSM guidelines has a significant intervention effect on SPPB.

The subgroup analysis findings revealed that the MD was 0.73 (95% CI: 0.01, 1.46) in the low- or unclear-consistency group, which was different (*p* = 0.05). This suggests that the SPPB intervention is impacted by exercise prescription that is inconsistent or poor in relation to ACSM standards.

**Figure 8:**
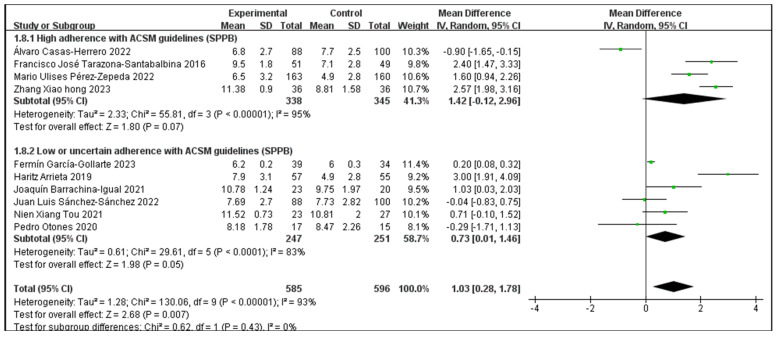
Forest plot for meta-analysis of the impact of exercise on SPPB indicators in frail elderly people [35,36,37,39,42,43,44,46,48,51].

In summary, exercise has a significant intervention effect on SPPB indicators, with an MD of 1.03 (95% CI: 0.28, 1.78) and (*p* = 0.007). Exercise prescriptions with low or uncertain ACSM guideline consistency had a more statistically significant impact on SPPB than those with high ACSM guideline consistency (*p* = 0.05 > *p* = 0.07). However, looking only at the mean of the intervention effect, the former is smaller than the latter (0.73 < 1.42). This suggests that exercise prescriptions with high consistency to ACSM guidelines may be more effective for SPPB intervention than those with low or uncertain consistency to ACSM guidelines (Figure 8). The higher heterogeneity in the two subgroups may be due to the intervention period, intervention measures, and study sample characteristics (Table 2). It should be noted that the higher the SPPB score, the better the physical function performance.

**Figure 9:**
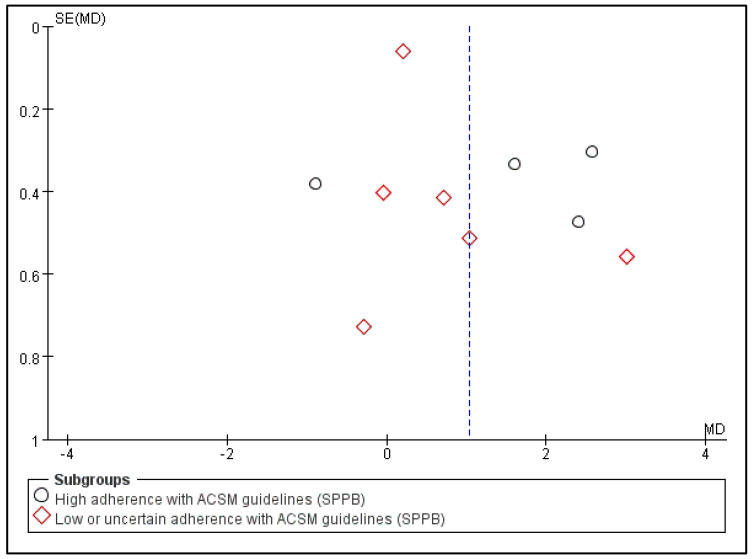
Funnel plot for SPPB indicator.

Subsequently, we conducted publication bias testing through REVIEW MANAGER 5.4.1. We observed the funnel plot and found that both sides were approximately symmetrical, indicating no obvious publication bias (Figure 9).

### 3.7. The Impact of Consistency with ACSM Recommendations on the Grip Strength

Outcome indicator 4 (grip strength) contains a total of 13 articles, with 712 people in the intervention group and 732 people in the control group; there was high consistency with ACSM guidelines in 7 articles, and low or uncertain consistency with ACSM guidelines in 6 articles. After the heterogeneity test (I^2^ = 78%), the random effects model was used for statistical analysis. All articles used the same scale for this outcome indicator, so mean difference (MD) was used for statistics and analysis.

Data analysis showed that the overall impact of exercise on the grip strength (GS) was 1.86 (95% CI: 0.75, 2.97), which was significantly different (*p* = 0.001). This shows that exercise has a significant intervention effect on GS.

The results of the subgroup analysis showed that in the high-consistency group, the MD was 2.39 (95% CI: 0.69, 4.09) and I^2^ = 74%, which was significantly different (*p* = 0.006). This indicates that exercise prescription with high consistency to ACSM guidelines significantly affects GS intervention.

The results of the subgroup analysis showed that in the low- or uncertain-consistency group, the MD was 1.1 (95% CI: −0.89, 3.09) and I^2^ = 84%, which was not significantly different (*p* = 0.28). It is unclear whether exercise prescription with low or uncertain consistency to ACSM guidelines has a significant intervention effect on GS.

**Figure 10:**
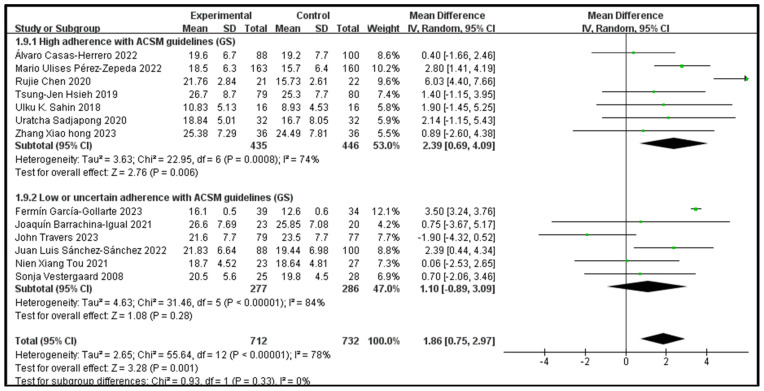
Forest plot for meta-analysis of the impact of exercise on GS indicators in frail elderly people [32,34,35,36,37,39,41,43,44,45,46,47,49].

In summary, exercise has a significant intervention effect on GS indicators; the MD was 1.86 (95% CI: 0.75, 2.97) and (*p* = 0.001). Exercise prescription with high consistency to ACSM guidelines has a more significant impact on GS than exercise prescription with low or uncertain consistency to ACSM guidelines (2.39 > 1.1), and the intervention effect is better. Because the GS score number is more significant, It indicates a lower degree of frailty [16] (Figure 10). The higher heterogeneity in the two subgroups may be due to the intervention period, intervention measures, and study sample characteristics (Table 2).

**Figure 11:**
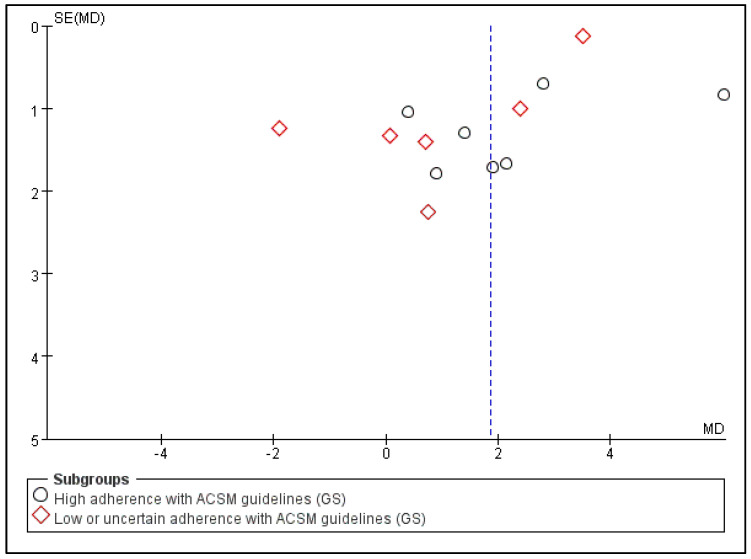
Funnel plot for GS indicator.

Subsequently, we conducted publication bias testing through REVIEW MANAGER 5.4.1. We observed the funnel plot and found that both sides were approximately symmetrical, indicating no obvious publication bias (Figure 11).

### 3.8. The Impact of Consistency with ACSM Recommendations on the Body Mass Index

There are three publications for outcome indicator 5 (BMI), with 89 participants in the intervention group and 83 participants in the control group. One study showed excellent consistency with ACSM standards, while the other two showed poor or questionable consistency (two articles). For statistical analysis, the fixed effects model was used after the heterogeneity test (I^2^ = 31%). Since this outcome variable was used on the same scale across all publications, statistics and analysis were performed using mean difference (MD).

Data analysis showed that the overall impact of exercise on body mass index (BMI) was −1.14 (95% CI: −2.17, −0.11), which was significantly different (*p* = 0.03). This shows that exercise has a significant intervention effect on BMI.

The results of the subgroup analysis showed that in the high-consistency group, the MD was −0.6 (95% CI: −1.84, 0.64), which was not significantly different (*p* = 0.34). This indicates that it is unclear whether exercise prescription with high consistency to ACSM guidelines significantly influences BMI.

The results of the subgroup analysis showed that in the low- or uncertain-consistency group, the MD was −2.37 (95% CI: −4.24, −0.5) and I^2^ = 0%, which was significantly different (*p* = 0.01). This indicates that exercise prescription with low or uncertain consistency to ACSM guidelines significantly affects BMI intervention.

**Figure 12:**
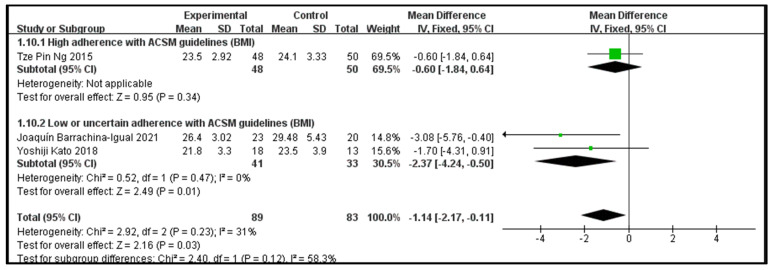
Forest plot for meta-analysis of the impact of exercise on BMI indicators in frail elderly people [37,38,50].

In summary, exercise significantly influences BMI indicators; the MD was −1.14 (95% CI: −2.17, −0.11) and (*p* = 0.03). Exercise prescription with low or uncertain consistency to ACSM guidelines has a more significant impact on BMI than exercise prescription with high consistency to ACSM guidelines (−2.37 > −0.6) (Figure 12). It is worth mentioning that BMI is a range index, and a noticeable intervention effect does not mean the actual impact is better.

**Figure 13:**
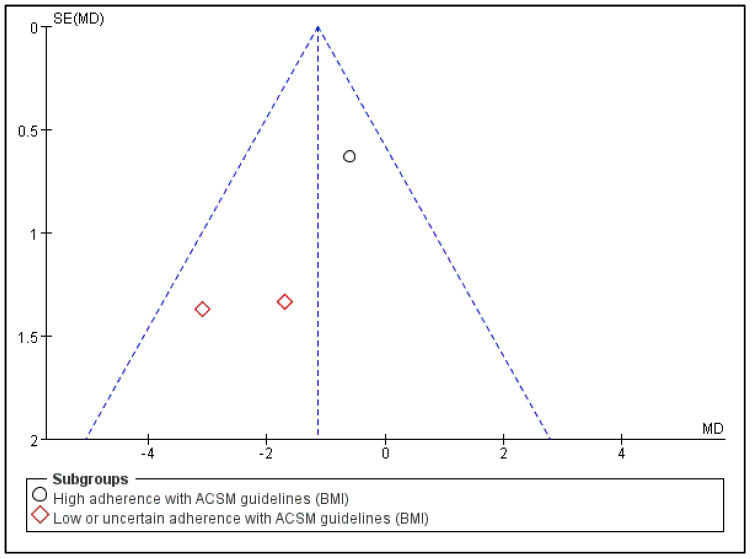
Funnel plot for BMI indicator.

Subsequently, we conducted publication bias testing through REVIEW MANAGER 5.4.1. We observed the funnel plot and found that both sides were approximately symmetrical, indicating no obvious publication bias (Figure 13).

## 4. Discussion

This study is based on the exercise prescriptions recommended by ACSM. It compares the effects of exercise prescriptions with high consistency and low or uncertain consistency recommended by ACSM on frailty in elderly people. A total of 20 studies were included, including 11 different exercise interventions and 2016 frail older adults. Exercise has once again proven to be a very effective intervention in improving frailty in elderly people [12]. It is worth mentioning that through meta-analysis, we found that exercise prescriptions with high consistency to ACSM recommendations have a significant intervention effect on two outcome indicators related to frailty in elderly people (Fried Frailty Phenotype and grip strength) and are better than exercise prescriptions with low or uncertain consistency to ACSM recommendations. However, exercise prescriptions with low or uncertain consistency to ACSM recommendations have a significant intervention effect on BMI, but this does not mean that there is an excellent actual effect. Finally, we found that exercise prescriptions with different consistency had no significant intervention effect on the two outcome indicators (Barthel Index, Short Physical Performance Battery). None of the 20 studies we included explicitly showed blinding of data analysts, so there is a risk of selection bias. It is important to note here that we did not assess the methodological quality and certainty of the included studies, so these viewpoints need to be viewed critically.

Exercise has a significant intervention effect on FP indicators. The requirements include five components: unintentional weight loss, fatigue, poor muscle strength, sluggishness, and physical inactivity [1]. First of all, it is well known that exercise has a direct intervention effect on these components, which is why both high consistency and low or uncertain consistency with the exercise prescription recommended by the ACSM can have a significant intervention effect on this indicator. Secondly, we found that the intervention effects differ depending on the frequency, intensity, and exercise duration. An ineffectively low dose will not impart full benefits, whereas the adverse effects stemming from an excessively high dose may overshadow potential benefits and introduce detriments [52]. This is also why high consistency to the exercise prescription recommended by the ACSM can improve this indicator’s intervention.

Through meta-analysis, we found that only exercise prescriptions with high consistency to ACSM recommendations have a significant intervention effect on this indicator (MD: 2.39, *p* = 0.006). It is worth mentioning that an increase in grip strength of more than 1.6 kg can be called a fundamental change [53]. Resistance training is considered an efficient treatment for age-related sarcopenia and can improve muscle strength and quality in patients [54], but it must also include grip strength. Therefore, a suitable resistance exercise program is essential. We believe that the resistance training program recommended by the ACSM is trustworthy because its training frequency, intensity, number of repetitions, and number of sets are similar to other resistance exercise programs for elderly people. These exercise prescriptions have been proven to be effective in improving the grip strength of elderly people [55,56,57] (Table 4).

Exercise has been shown to improve ADL, which is consistent with the results of our meta-analysis (MD: 5.79, *p* = 0.02). However, whether it is high or low or uncertain consistency with the exercise prescription recommended by the ACSM, the intervention effect on BI indicator is insignificant (*p* = 0.06, *p* = 0.14). The intervention effect of exercise on SPPB indicator is also significant (MD: 1.03, *p* = 0.007). However, whether it is high or low or uncertain consistency with the exercise prescription recommended by the ACSM, the intervention effect on this indicator is insignificant (*p* = 0.07, *p* = 0.05). We speculate that this may be because the BI and SPPB indicators do not lead to apparent differences in intervention effects due to specific exercise doses. This is why they are widely used as secondary outcome indicators in some exercise dosage experiments. Exercise has a significant intervention effect on BMI (MD: −1.14, *p* = 0.03). However, only the intervention effect of high consistency to ASCM recommended exercise prescription is not substantial (*p* = 0.34). This may be because only one article in the high-consistency group includes this indicator. In addition, BMI is only a statistical index used to estimate body fat; the impact of obesity on health outcomes in elderly people is complex; this has been described as the obesity paradox [58]. This is also why most experiments use BMI as a baseline measurement or secondary outcome indicator.

In summary, although exercise has a significant intervention effect on five outcome indicators related to frailty in elderly people, exercise prescriptions with high consistency to ACSM recommendations have a more substantial impact on two frequently used primary outcome indicators (Fried Frailty Phenotype, grip strength). Different exercise doses did not affect the differences in the intervention effects of the remaining three outcome indicators.

## 5. Strengths and Limitations

Firstly, the topic of our meta-analysis is novel and has high clinical significance. Currently, many studies focus on the intervention effects of different exercise prescriptions on frailty in elderly people. The most critical part of the intervention in these studies was the dose of exercise [59]. However, many studies or scholars have disagreed on how much exercise should be given to pre-frail or frail older adults. Of course, this is also because people consider the clinical value of personalized exercise prescription [60]. Nonetheless, the standardized application of exercise dose is still a topic worthy of discussion.

Secondly, our meta-analysis has the following limitations: 1: The number of articles included in the final study is small, although we tried to obtain as many articles as possible through various methods. We did not search the databases SCOPUS, CINAHL, PEDro, LILACS, and some gray literature; 2: The research results are interfered with by some confounding factors (age, gender, weight, physical condition, etc.), so it is difficult for us to avoid heterogeneity between studies; 3: We did not assess the quality and certainty of the included studies; 4: We did not test for MICD (minimum significant clinical difference).

Finally, readers need to be cautious when interpreting our findings, especially among those outcomes with small sample sizes.

## 6. Conclusions

This review again supports and demonstrates that exercise can improve frailty in older adults. In exploring the optimal exercise dose for older adults with pre-frailty and frailty, we found that exercise prescriptions with high consistency with ACSM recommendations may be more effective as reflected in the outcome measures of FP and GS than for those with uncertain or low consistency. However, it is essential to note that the data derived from the meta-analysis is still subject to the small number of studies, the unknown degree of adherence to the exercise of the participants in individual studies, and the different mix of cases in the studies. There is, therefore, a need to expand the sample size in this field to verify the impact of interventions on the five outcomes related to frailty in elderly people. In addition, there are many methodological biases in the evidence based on the included interventions, so the above conclusions can only be preliminary.

## Figures and Tables

**Table 1:** The ACSM guidelines for muscular strength, flexibility, and cardiorespiratory fitness in individuals who seem to be in good health.

Exercise Dose	Cardiorespiratory Exercise	Resistance Exercise	Flexibility Exercise
Frequency	4–5 days/week	On non-consecutive days, 1–2 days each week, progressively rising to 2–3 days per week.	5–7 days/week
Intensity/workload	Moderate intensity, 40–59%VO^2^R/HRR, CR-10 scale rating of 3–4	The last two sets should be difficult; adjust the resistance accordingly. If it’s manageable, high-intensity exercise may be done.	Stretch until you feel your muscles being pulled tight or a slight discomfort.
Duration	Gradually increase from 20 min to at least 30 min (up to 45–60 min)	After approximately two weeks, work your way up to two sets of eight to twelve repetitions. Don’t exceed 8–10 exercises in a single session.	Repeated 2–4 times, static stretching is sustained for 10–30 s.

HRR: heart rate reserve. VO^2^R: oxygen uptake reserve.

**Table 2:** Included article characteristics.

Author	Country	Year	Population	Age (Mean + SD)	Total/Male/Female	Intervention	Control	Outcome
Juan Luis Sánchez-Sánchez [39]	France	2022	Frailty Pre-frailty	T:84.15(4.76) C:83.99(4.8)	T:88/28/60 C:100/40/60	Vivifrail training Length of Intervention: 12 weeks Freq: 3–5 times a week Duration: 30–60 min	CON	SPPB Grip strength
Ulku K. Sahin [34]	Turkey	2018	Frailty	T:84.18(6.85) C:85.37(4.7)	T:16/NA/NA C:16/NA/NA	Resistance training Length of Intervention: 8 weeks Freq: 3 times a week Duration: 40 min	CON	Grip strength BI
Haritz Arrieta [42]	Spain	2019	Frailty	T:85.1(7.6) C:84.7(6.1)	T:57/15/42 C:55/18/37	MEP Length of Intervention: 24 weeks Freq: 2 times a week Duration: 60 min	CON	SPPB BI
Maria Giné-Garriga [33]	Spain	2010	Frailty	T:83.9(2.8) C:84.1(3)	T:22/9/13 C:19/7/12	FCT Length of Intervention: 12 weeks Freq: 2 times a week Duration: 45 min	CON	BI
Sonja Vestergaard [45]	Denmark	2008	Frailty	T:81(3.3) C:82.7(3.8)	T:25/0/25 C:28/0/28	Vivifrail training Length of Intervention: 12 weeks Freq: 3–5 times a week Duration: 30–60 min	CON	Grip strength
Yoshiji Kato [38]	Japan	2018	Frailty	T:77.6(7.2) C:79.6(7.7)	T:18/11/7 C:13/7/6	NHK&MP&CR Length of Intervention: 12 weeks Freq: 7 times a week Duration: 25 min	CON	BMI BI
Francisco José Tarazona-Santabalbina [48]	Spain	2016	Frailty	T:79.7(3.6) C:80.3(3.7)	T:51/22/29 C:49/24/25	MEP Length of Intervention: 24 weeks Freq: 5 times a week Duration: 65 min	CON	SPPB BI Fried
Tsung-Jen Hsieh [47]	Taiwan	2019	Frailty Pre-frailty	T:72.0(6.0) C:72.5(5.5)	T:79/28/60 C:80/40/60	Exercise training Length of Intervention: 24 weeks Freq: 3–7 times a week Duration: 5–60 min	CON	Fried Grip strength
Tze Pin Ng [50]	Singapore	2015	Frailty Pre-frailty	T:70.3(5.25) C:70.1(5.02)	T:48/21/27 C:50/22/28	Physical training Length of Intervention: 12 weeks Freq: 2 times a week Duration: 90 min	CON	BMI Fried
Álvaro Casas-Herrero [43]	Spain	2022	Frailty	T:84.2(4.8) C:84.0(4.8)	T:88/25/63 C:100/31/69	Vivifrail training Length of Intervention: 12 weeks Freq: 5 times a week Duration: 30 min	CON	SPPB BI Grip strength
John Travers [32]	Ireland	2023	Frailty	T:77.6(5.2) C:76.5(5.2)	T:79/25/54 C:77/26/51	Home-based exercise Length of Intervention: 12 weeks Freq: 4 times a week Duration: 45–60 min	CON	Grip strength
Mario Ulises Pérez-Zepeda [46]	Canada	2022	Frailty	T:87.1(4.8) C:87.9(4.5)	T:163/72/91 C:160/69/91	Vivifrail training Length of Intervention: 12 weeks Freq: 3–5 times a week Duration: 30–60 min	CON	BI SPPB Grip strength
Rujie Chen [41]	China	2020	Pre-frailty	T:76.97(5.19) C:75.27(5.98)	T:33/12/21 C:33/11/22	Elastic band training Length of Intervention: 8 weeks Freq: 3 times a week Duration: 45–60 min	CON	Grip strength
T. Liu [40]	China	2022	Frailty	T:80.75(2.99) C:80.74(2.82)	T:67/16/51 C:68/24/44	Integrated exercise Length of Intervention: 12 weeks Freq: 5 times a week Duration: 40 min	CON	Fried
Zhang Xiaohong [44]	China	2023	Frailty	T:64.75(4.35) C:64.94(4.29)	T:36/13/23 C:36/20/16	Dance training Length of Intervention: 16 weeks Freq: 5 times a week Duration: 60 min	CON	SPPB Grip strength
Joaquín Barrachina-Igual [37]	Spain	2021		T:74.83(5.78) C:75.25(8.20)	T:23/7/16 C:20/5/15	MEP Length of Intervention: 12 weeks Freq: 2 times a week Duration: 65 min	CON	BI SPPB Grip strength
Nien Xiang Tou [36]	Singapore	2021	Frailty	T:79.5(4.2) C:74.6(6.5)	T:23/NA/NA C:27/NA/NA	FPT Length of Intervention: 12 weeks Freq: 2 times a week Duration: 60 min	CON	Fried SPPB Grip strength
Pedro Otones [51]	Spain	2020	Frailty	T:79.5(4.2) C:74.6(6.5)	T:17/5/12 C:15/2/13	Physical training Length of Intervention: 32 weeks Freq: 1 time a week Duration: 60 min	CON	SPPB Grip strength
Uratcha Sadjapong [49]	Thailand	2020	Frailty	T:76.68(1.14) C:78.87(1.32)	T:32/16/16 C:32/23/9	MEP Length of Intervention: 12 weeks Freq: 3 times a week Duration: 60 min	CON	Fried Grip strength
Fermín García-Gollarte [35]	Spain	2023	Frailty	T:86(5.9) C:84.9(6)	T:39/9/30 C:34/13/21	OEP training Length of Intervention: 24 weeks Freq: 3 times a week Duration: 40–60 min	CON	SPPB Grip strength

Note: SD: standard deviation, CON: control group with routine care (no exercise), T: experimental group, C: control group; MEP: multi-component exercise program, FCT: functional circuit training, NHK: NHK radio calisthenics, MP: marching in place, CR: chair rising. FPT: functional power training, OEP: Otago Exercise Program. SPPB: Short Physical Performance Battery, BI: Barthel Index, BMI: body mass index, Fried: Fried scale.

**Table 3:** Exercise interventions evaluated according to the American College of Sports Medicine’s (ACSM) recommendations.

Author, Year	Cardiorespiratory Exercise						Resistance Exercise								Flexibility Exercise						ACSM Consistency
	Frequency		Intensity/Workload		Duration		Frequency		Intensity/Workload		Repetitions		Sets		Frequency		Intensity/Workload		Duration		Points (Percent)
	4–5 d/wk		40–59% VO2R/HRR, Moderate Exertion, with a CR-10 Scale Grade of 3–4.		Increase by 20 Minutes Gradually to at Least 30 Min (or Up to 45–60 Min).		1–2 Days per Week, Rising to 2–3 Days per Week over Time		Change the Resistance from Medium to High.		8–12		1–2		5–7 d/wk		Stretch until You Start to Feel a Mild Soreness or a Tugging Sensation in Your Muscles.		Repeated 2–4 Times, Static Stretching Is Sustained for 10–30 s.		
Juan Luis Sánchez-Sánchez, 2022 [39]	5	☺	Walk at the usual pace.	☺	50–105 (s)	☹	3	☺	30 RM	☹	12–15	☹	2	☺	3	☹	NR	☹	20–30 (s)	☺	11/20 (55%)
Ulku K. Sahin, 2018 [34]							3	☺	40–70% 1 RM	☺	6–10	☺	NR	☹							7/8 (88%)
Haritz Arrieta, 2019 [42]	7	☹	NR	☹	20 min	☺	2	☺	40–70% 1 RM	☺	NR	☹	NR	☹	2	☹	NR	☹	5 (min)	☹	10/20 (50%)
Maria Giné-Garriga, 2010 [33]	2	☹	Walk at the usual pace.	☺	10 min	☹	2	☺	12–14 RM	☺	6–15	☺	1–2	☺	2	☹	NR	☹	5 (min)	☹	11/20 (55%)
Sonja Vestergaard, 2008 [45]	3	☹	Walking on the spot	☹	5 min	☹	3	☺	NR	☹	NR	☹	NR	☹	3	☹	NR	☹	15 (min)	☹	6/20 (30%)
Yoshiji Kato, 2018 [38]	7	☹	Walking on the spot	☹	15 min	☹	7	☹	NR	☹	NR	☹	NR	☹	7	☺	NR	☹	5 (min)	☹	6/20 (30%)
Francisco José Tarazona-Santabalbina, 2016 [48]	3	☹	Moderate intensity	☺	40 min	☺	2	☺	75%1 RM	☺	8	☺	3	☹	5	☺	NR	☹	20 (s)	☺	15/20 (75%)
Tsung-Jen Hsieh, 2019 [47]							3–7	☹	ACSM intensity	☺	8–12	☺	1–2	☺	3–7	☹	ACSM intensity	☺	10–30 s	☺	12/14 (86%)
Tze Pin Ng, 2015 [50]							2	☺	ACSM intensity	☺	8–15	☺	NR	☹							7/8 (88%)
Álvaro Casas-Herrero, 2022 [43]	5	☺	Walk at the usual pace.	☺	24 min	☺	3	☺	NR	☹	12	☺	3	☹	3	☹	NR	☹	10 s	☺	14/20 (70%)
John Travers, 2023 [32]	3–4	☹	Walking	☺	35–45 min	☺	4–7	☹	NR	☹	10–15	☺	≥4	☹							8/14 (57%)
Mario Ulises Pérez-Zepeda, 2022 [46]	NR	☹	Walk at the usual pace.	☺	24 min	☺	NR	☹	30–60% 1 RM	☹	8–10	☺	2–3	☺							10/14 (71%)
Rujie Chen, 2020 [41]							3	☺	NR	☹	10–15	☺	2	☺							7/8 (88%)
T. Liu, 2022 [40]	5	☺	Tai Chi	☺	40 min	☺									5	☺	NR	☹	NR	☹	10/12 (83%)
Sonja Vestergaard, 2008 [45]	5	☺	Dance	☺	40 min	☺									5	☺	NR	☹	NR	☹	10/12 (83%)
Yoshiji Kato, 2018 [38]	2	☹	Walking	☺	10 min	☹	2	☺	≥70%1 RM	☺	10–15	☺	3	☹	2	☹	NR	☹	NR	☹	10/20 (50%)
Francisco José Tarazona-Santabalbina, 2016 [48]							2	☺	NR	☹	10–20	☺	3	☹							5/8 (63%)
Tsung-Jen Hsieh, 2019 [47]							NR	☹	NR	☹	NR	☹	NR	☹							4/8 (50%)
Tze Pin Ng, 2015 [50]	≥3	☺	40–65% HRR	☹	10–20 min	☹	≥3	☹	65–100%1 RM	☺	8–12	☺	2–3	☺							10/14 (71%)
Álvaro Casas-Herrero, 2022 [43]	NR	☹	NR	☹	NR	☹	NR	☹	NR	☹	NR	☹	NR	☹							7/14 (50%)

ACSM: American College of Sports Medicine. NR: not reported. Happy/green face: meets the recommendations (2 points), neutral/yellow face: not sure if the recommendations are met (1 point), unhappy/red face: does not meet the recommendations (0 points).

**Table 4:** Comparison of ACSM recommended exercise prescriptions and other exercise prescriptions in resistance exercise.

	Frequency	Intensity/Workload	Repetitions	Sets
ACSM exercise prescriptions	1–2 d/wk, 2–3 d/wk	Change the resistance from medium to high	8–12	1–2
Other exercise prescriptions (1)	2–3 d/wk	1 RM 50–80%	5–8	1–2
Other exercise prescriptions (2)	2 d/wk	1 RM 51–69%	7–9	2–3
Other exercise prescriptions (3)	2 d/wk	relatively high degree of effort	6–12	1–3

Subsequently, we found that exercise prescriptions with low or uncertain consistency to ACSM recommendations were not detailed enough when designing resistance exercise programs (Table 3), which resulted in insignificant intervention effects for patients (MD: 1.1, *p* = 0.28).

## Data Availability

The original contributions presented in the study are included in the article/Supplementary Materials; further inquiries can be directed to the corresponding author.

## Supplementary Materials

The following supporting information can be downloaded at: https://www.mdpi.com/article/10.3390/jcm13113037/s1, Table S1: Search Strategy.
