# Supplementary material for: Effects of Exercise on Frailty in Older People Based on ACSM Recommendations: A Systematic Review and Meta-Analysis of Randomized Controlled Trials
Source: J Clin Med. 2024 May 22;13(11):3037. doi: 10.3390/jcm13113037 (PMC11173309; doi:10.3390/jcm13113037)
Supplement: Supplementary file 1 [file jcm-13-03037-s001.zip › jcm-2963199-supplementary.pdf]

Supplementary Material

Table S1. Search Strategy

Database	Search strategy	amount
	PubMed	
#1	Search: (Asthenia[MeSH Terms]) OR (Frailty[MeSH Terms])	11954
#2	Search: ((((((((Asthenia[Title/Abstract]) OR (Frailty[Title/Abstract])) OR (Fatigue[Title/Abstract])) OR (Neurasthenia[Title/Abstract])) OR (Muscle Weakness[Title/Abstract])) OR (Frailties[Title/Abstract])) OR (Frailness[Title/Abstract])) OR (Frailty Syndrome[Title/Abstract])) OR (Debility[Title/Abstract])) OR (Debilities[Title/Abstract])	181202
#3	#1 OR #2	182671
#4	Search: ((Exercise[MeSH Terms]) OR (walking[MeSH Terms])) OR (Nordic Walking[MeSH Terms])	277004
#5	Search: ((((((((((((((((((((((((((Exercise[Title/Abstract]) OR (walking[Title/Abstract])) OR (Nordic Walking[Title/Abstract])) OR (Exercises[Title/Abstract])) OR (Physical Activity[Title/Abstract])) OR (Activities, Physical[Title/Abstract])) OR (Activity, Physical[Title/Abstract])) OR (Physical Activities[Title/Abstract])) OR (Exercise, Physical[Title/Abstract])) OR (Exercises, Physical[Title/Abstract])) OR (Physical Exercise[Title/Abstract])) OR (Physical Exercises[Title/Abstract])) OR (Exercise, Aerobic[Title/Abstract])) OR (Aerobic Exercise[Title/Abstract])) OR (Aerobic Exercises[Title/Abstract])) OR (Exercises, Aerobic[Title/Abstract])) OR (Exercise Training[Title/Abstract])) OR (Exercise Trainings[Title/Abstract])) OR (Training, Exercise[Title/Abstract])) OR (Trainings, Exercise[Title/Abstract])) OR (Training, Resistance[Title/Abstract])) OR	828397

	(Strength Training[Title/Abstract])) OR (Training, Strength[Title/Abstract])) OR (Balance[Title/Abstract])) OR (Ambulation[Title/Abstract])) OR (Stair Climbing[Title/Abstract])) OR (Walking, Nordic[Title/Abstract])) OR (Pole Walking[Title/Abstract])) OR (Walking, Pole[Title/Abstract])	
#6	#4 OR #5	907666
#7	Search: ((((Randomized controlled trial[Publication Type]) OR (controlled clinical trial[Publication Type])) OR (randomized[Title/Abstract])) OR (placebo[Title/Abstract])) OR (randomly[Title/Abstract])	1372002
#8	Search: aged[MeSH Terms]	3479861
#9	Search: (elderly[Title/Abstract]) OR (aged[Title/Abstract])	997981
#10	(#8) OR (#9)	4059909
#11	#3 AND #6 AND #7 AND #10	2027
	Embase	
#1	asthenia:ab,ti OR frailty:ab,ti OR fatigue:ab,ti OR neurasthenia:ab,ti OR 'muscle weakness':ab,ti OR frailties:ab,ti OR frailness:ab,ti OR 'frailty syndrome':ab,ti OR debility:ab,ti OR debilities:ab,ti	286165
#2	(exercise:ab,ti OR walking:ab,ti OR 'nordic walking':ab,ti OR exercises:ab,ti OR 'physical activity':ab,ti OR 'activities, physical':ab,ti OR 'activity, physical':ab,ti OR 'physical activities':ab,ti OR 'exercise, physical':ab,ti OR 'exercises, physical':ab,ti OR 'physical exercise':ab,ti OR 'physical exercises':ab,ti OR 'exercise, aerobic':ab,ti OR 'aerobic exercise':ab,ti OR 'aerobic exercises':ab,ti OR 'exercises, aerobic':ab,ti OR 'exercise training':ab,ti OR 'exercise trainings':ab,ti) AND 'training, exercise':ab,ti AND 'trainings, exercise':ab,ti AND 'training, resistance':ab,ti AND 'strength training':ab,ti OR 'training, strength':ab,ti OR balance:ab,ti OR ambulation:ab,ti OR 'stair climbing':ab,ti OR 'walking, nordic':ab,ti OR 'pole walking':ab,ti OR 'walking, pole':ab,ti	379394
#3	'controlled clinical trial':ab,ti OR 'randomized controlled trial':ab,ti	165482
#4	aged:ab,ti OR elderly:ab,ti	1370078

#5	#1 AND #2 AND #3 AND #4	45
	Web of Science	
#1	((((((((ALL=(Asthenia)) OR ALL=(Frailty)) OR TS=(Fatigue)) OR TS=(Neurasthenia)) OR TS=(Muscle Weakness)) OR TS=(Frailties)) OR TS=(Frailness)) OR TS=(Frailty Syndrome)) OR TS=(Debility)) OR TS=(Debilities)	389887
#2	((((((((((((((((((((((((((ALL=(Exercise)) OR ALL=(walking)) OR ALL=(Nordic Walking)) OR TS=(Exercises)) OR TS=(Physical Activity)) OR TS=(Activities, Physical)) OR TS=(Activity, Physical)) OR TS=(Physical Activities)) OR TS=(Exercise, Physical)) OR TS=(Exercises, Physical)) OR TS=(Physical Exercise)) OR TS=(Physical Exercises)) OR TS=(Exercise, Aerobic)) OR TS=(Aerobic Exercise)) OR TS=(Aerobic Exercises)) OR TS=(Exercises, Aerobic)) OR TS=(Exercise Training)) OR TS=(Exercise Trainings)) OR TS=(Training, Exercise)) OR TS=(Trainings, Exercise)) OR TS=(Training, Resistance)) OR TS=(Strength Training)) OR TS=(Training, Strength)) OR TS=(Balance)) OR TS=(Ambulation)) OR TS=(Stair Climbing)) OR TS=(Walking, Nordic)) OR TS=(Pole Walking)) OR TS=(Walking, Pole)	2177119
#3	((((TS=(Randomized controlled trial)) OR TS=(controlled clinical trial)) OR TS=(randomized)) OR TS=(placebo)) OR TS=(randomly)	1695043
#4	(TS=(aged)) OR TS=(elderly)	4293505
#5	#1 AND #2 AND #3 AND #4	3712
	Cochrane	
#1	(Asthenia):ti,ab,kw OR (Frailty):ti,ab,kw OR (Fatigue):ti,ab,kw OR (Neurasthenia):ti,ab,kw OR (Muscle Weakness):ti,ab,kw	55303
#2	(Frailties):ti,ab,kw OR<br>(Frailness):ti,ab,kw OR<br>(Frailty Syndrome):ti,ab,kw OR<br>(Debility):ti,ab,kw OR (Debilities):ti,ab,kw	5643
#3	#1 OR #2	57366
#4	Exercise):ti,ab,kw OR<br>(walking):ti,ab,kw OR<br>(Nordic Walking):ti,ab,kw OR<br>(Exercises):ti,ab,kw OR (Physical Activity):ti,ab,kw	198100
#5	(Activities, Physical):ti,ab,kw OR (Activity, Physical):ti,ab,kw OR (Physical Activities):ti,ab,kw OR	97397

	(Exercise, Physical):ti,ab,kw OR (Exercises, Physical):ti,ab,kw	
#6	(Physical Exercise):ti,ab,kw OR (Physical Exercises):ti,ab,kw OR (Exercise, Aerobic):ti,ab,kw OR (Aerobic Exercise):ti,ab,kw OR (Aerobic Exercises):ti,ab,kw	65090
#7	(Exercises, Aerobic):ti,ab,kw OR (Exercise Training):ti,ab,kw OR (Exercise Trainings):ti,ab,kw OR (Training, Exercise):ti,ab,kw OR (Trainings, Exercise):ti,ab,kw	59375
#8	(Training, Resistance):ti,ab,kw OR (Strength Training):ti,ab,kw OR (Training, Strength):ti,ab,kw OR (Balance):ti,ab,kw OR (Ambulation):ti,ab,kw	85345
#9	(Stair Climbing):ti,ab,kw OR (Walking, Nordic):ti,ab,kw OR (Pole Walking):ti,ab,kw OR (Walking, Pole):ti,ab,kw	2047
#10	#4 OR #5 OR #6 OR #7 OR #8 OR #9	243793
#11	(Randomized controlled trial):ti,ab,kw OR (controlled clinical trial):ti,ab,kw OR (randomized):ti,ab,kw	1356512
#12	(aged):ti,ab,kw OR (elderly):ti,ab,kw	885950
#13	#3 AND #10 AND #11 AND #12	8401
